# Bernoulli mixture models in application to the evaluation of algorithms estimating functionality of missense mutations

**DOI:** 10.1186/1753-6561-6-S6-P15

**Published:** 2012-10-01

**Authors:** Stephanie Hicks, Sharon E Plon, Marek Kimmel

**Affiliations:** 1 Department of Statistics, Rice University, Houston, TX, USA; 2 Departments of Pediatrics and Human and Molecular Genetics, Baylor College of Medicine, Houston, TX, USA

## Background

Whole genome and whole exome sequencing projects yield thousands of missense mutations with unknown functionality. Direct estimation of the sensitivity and specificity of bioinformatic algorithms predicting the impact of missense mutations on protein function requires a 'gold standard' or set of mutations with known functionality. In the absence of a gold standard, additional statistical methods are needed to estimate the accuracy of these algorithms. It has been shown informative predictions depend on the algorithm and sequence alignment employed and often algorithms disagree as to which mutations are predicted deleterious or neutral [1].

## Materials and methods

To investigate the level of agreement, disjoint categories of sets of mutations are defined depending on which algorithms predict which mutations to be deleterious or neutral. We have developed two statistical models called Bernoulli mixture (BM) and augmented Bernoulli mixture (ABM) based on the capture-recapture technique that employs these disjoint categories. Application of these models allows us to jointly estimate the sensitivities and specificities of each algorithm considered without the use of a gold standard and to estimate the proportion of deleterious mutations in a given set. These estimates may then be used to calculate the posterior probability of a given variant being deleterious. When considering *n* algorithms, there are 2" disjoint categories employed by the ABM model, which includes 2*n* + 3 parameters, and the BM model is a special case of the ABM model that includes 2*n* + 1 parameters. We use the expectation-maximization algorithm for parameter estimation.

## Results

We apply the models to two types of predictions of functionality: simulated and real predictions. Using simulated predictions, we accurately recover the true sensitivity and specificity values and report confidence regions. We show example posterior probabilities of a given variant being deleterious. When a gold standard is available, we show the sensitivity and specificity estimates reported the BM and ABM models closely match the sensitivity and specificity estimated directly using the true functionality status. To test our models on mutations without known functionality, we apply the models to mutations obtained from the exomes of four individuals which were sequenced at the Human Genome Sequencing Center at Baylor College of Medicine to identify cancer susceptibility genes for acute lymphocytic leukemia and lymphoma in children. Within each individual, we estimate posterior probabilities for each variant being deleterious and apply an intersection filter to look for deleterious mutations shared by the three affected individuals, but not in the unaffected individual.

## Conclusions

The BM and ABM models may be used to estimate the sensitivity and specificity of algorithms predicting the functionality of mutations without the use of a gold standard and to calculate posterior probabilities of a given variant being deleterious which may be used downstream in application of finding causal variants in next-generation sequencing.
